# Prospective Association Between Tobacco Use and at-Risk Alcohol Consumption Among Swedish Adolescents: Outlining the Influence of Tobacco Product, Frequency of Use and Gender in the LoRDIA Cohort

**DOI:** 10.1177/1179173X241298524

**Published:** 2024-10-30

**Authors:** Johanna Andersson, Kristina Berglund, Robin Irmel, Louise Adermark

**Affiliations:** 1 Department of Pharmacology, Institute of Neuroscience and Physiology, The Sahlgrenska Academy, 195564 University of Gothenburg, Gothenburg, Sweden; 2 Institute of Psychology, The Faculty of Social Sciences, 195564 University of Gothenburg, Gothenburg, Sweden

**Keywords:** adolescence, Alcohol Use Disorders Identification Test, ethanol, nicotine, oral nicotine, smokeless tobacco, smoking, snus

## Abstract

**Introduction:** Tobacco use is not only a major risk factor for morbidity and mortality but also associated with alcohol misuse. While personality traits may be driving this association, the psychoactive component of tobacco, nicotine, may also be a major risk factor. The aim with this study was to further assess the prospective association between tobacco use and alcohol consumption, with special emphasis on the role of the tobacco product used (cigarettes and Swedish snus), frequency of use, and gender. **Methods:** Data was extracted from the prospective cohort Longitudinal Research on Development In Adolescence (LoRDIA), following Swedish adolescents over four waves (∼13 to 17 years of age). Tobacco use was reported with respect to product used and frequency of use, while alcohol use was assessed using AUDIT-C, as well as frequency of use within the last year. **Results:** Use of tobacco, independent of product used and gender, was associated with increased alcohol consumption. High frequency of use and dual use strengthened to association. Individuals initiating tobacco use during the study period progressively increased their frequency of alcohol consumption compared to non-users during consecutive waves. Furthermore, tobacco use was associated with at-risk consumption of alcohol at follow up, even when adjusting for previous alcohol inebriation, socioeconomical factors, gender and novelty seeking. **Conclusions:** The data presented here suggests that nicotine use during adolescence, and especially dual use, is a major risk factor for future hazardous alcohol intake. This finding is especially important considering the escalated use of nicotine pouches, which in many ways resembles Swedish snus. From a public health perspective, preventive measures and policies designed to counteract all forms of nicotine use among youths is warranted.

## Introduction

Adolescence is a developmental period characterized by increased reward-seeking behavior, and experimentation with substance use, including nicotine and alcohol.^1-3^ While the adolescent brain is especially susceptible for the rewarding and reinforcing properties of drugs of abuse,^4-6^ smoking in adolescence presents a unique set of health-risks.^7,8^ Indeed, as the brain is still maturing, it is also more vulnerable for nicotine-induced neuroplasticity.^5,9,10^ Especially, nicotine use during adolescence has been postulated to transform brain reward circuits,^9,11,12^ and to worsen cognitive and attentional performance.^13,14^ These neurophysiological transformations may partially explain why smoking initiation at a young age decreases the likelihood of cessation,^15,16^ but could also contribute to the increased likelihood of using other drugs of abuse, including alcohol.^7,17^

There is a well-established association between nicotine use and hazardous alcohol intake,^18-20^ and adolescent smokers demonstrate increased vulnerability to alcohol use disorder (AUD) compared with nonsmokers who drink equivalent amounts of alcohol.^7^ While genetics and personality traits may explain parts of these associations,^21^ experimental studies in animal models support a causal relationship. In fact, the psychoactive component in tobacco, nicotine, appears to affect the rewarding effects by alcohol through activation of nicotinergic acetylcholine receptors (nAChRs).^22,23^ In addition, repeated exposure to nicotine increases alcohol consumption in experimental animals,^24,25^ whereas the partial nAChR agonist varenicline decreases alcohol consumption in both humans and rodents.^26,27^ However, if any form of tobacco use may increase the likelihood of alcohol use, and if the association is dependent on gender, has not been fully outlined.

Behaviors associated with tobacco use partially depend on gender, with reported differences in prevalence of use, sensitivity to nicotine reinforcement, and success rates in tobacco cessation.^28,29^ While men seem to be more sensitive to the rewarding effects of nicotine,^30^ women report smoking for stress relieve and affect regulation.^31^ Furthermore, while male gender is associated with higher rates of alcohol consumption, binge drinking and dependence, these differences tend to emerge later in life.^32-34^ Whether there is a gender influence with respect to the association between tobacco initiation and alcohol consumption in adolescence has not been fully defined.

While smoking repeatedly has been linked to alcohol drinking,^7,35,36^ less is known about smokeless tobacco products such as Swedish snus. Although smokeless tobacco may increase the risk for smoking initiation^37^ and alcohol use,^38,39^ few studies have outlined the association, and longitudinal studies are scarce. Importantly, since the launch of nicotine pouches, which in many ways resemble Swedish snus, dual use of nicotine products is on the rise. While polytobacco use among cigarette smokers is associated with reduced cigarette craving,^40^ smokeless tobacco does not appear to increase the chances of nicotine cessation.^41,42^ Furthermore, use of several nicotine products may increase the risk of other substance use, including alcohol.^40^ Understanding the association between Swedish snus and alcohol misuse, and the impact of dual product use, is thus important to increase awareness regarding risk factors associated with the use of non-combustible nicotine.

Based on the intricate association between nicotine use and alcohol misuse, and the escalating use of smokeless nicotine products among youths, the aim of this study was to define the prospective association between tobacco use and alcohol consumption among Swedish adolescents. Especially we wanted to explore the influence displayed by the form of tobacco product used (combustible or non-combustible), frequency of use and gender.

## Methods

### Study design

In order to outline the longitudinal relationship between tobacco use and alcohol consumption we extracted relevant data from the Longitudinal Research on Development In Adolescence (LoRDIA) program, which follows Swedish adolescents over four years.^43^ Especially we were interested in defining if the Swedish smokeless tobacco product *snus*, would have similar alcohol intake enhancing properties as demonstrated for smoking, and if the effects by tobacco differed between gender. Tobacco use was further stratified based on age at initiation, frequency of use and dual use. Alcohol use was assessed with regards to alcohol intake, inebriation and AUDIT-C score. Logistic regressions were adjusted for personality traits, socioeconomic factors and inebriation during earlier waves.

### Participants and procedure

This study is based on the ongoing prospective longitudinal program LoRDIA which focuses on social, behavioral and psychological developmental trajectories in a general population.^43^ In 2013, all children born in four Swedish cities in 2001 were invited to participate in the study. There were no exclusion criteria, and the program also included children with disabilities. The adolescents were recruited from four towns and 15 different schools in the south of Sweden. Both the children and their parents were provided with written information about the project, and participation in the study required written consent from both the children and the parents. Data was collected via surveys in schools. There was always a research assistant available at school to answer questions and help children with disabilities. Data collection was initiated in 2013, and the data in the present study was collected when the adolescents were in mean 13 to 17 years of age (Table 1). The project has been approved by the Regional Research Review Board in Gothenburg (No. 362-13; 2013-09-25; 2014-05-20; 2015-09-02).

**Table 1.** Demographics of Study Participants Study. Sample Characteristics Throughout the Four Study Waves.

Participants	W1	W2	W3	W4
Number of study participants (n)	1515	1454	1322	949
Gender (%)				
Girls	51	52	51	56
Boys	49	48	49	44***
Mean age (years)	12.59 ± 0.64	13.35 ± 0.62	14.32 ± 0.64	16.95 ± 0.44
Novelty seeking (mean ±sd)				
Total		7.8 ± 3.4		
Girls		7.5 ± 3.4		
Boys		8.1 ± 3.3***		
Self-rated socioeconomic status (%)				
Less money	11	7	7	8
As much	70	80	77	74
More money	19	13	16	18
Individuals lost to follow up		**W2**	**W3**	**W4**
% of participants in previous wave		16	19	43
% of tobacco users / non-users		20 / 16	28 / 18***	54 / 40***

***P < 0.001

#### Exposure variables

##### Tobacco use

At baseline (W1), questions about cigarette and snus use addressed ever use (yes/no), and current use (five grade ordinal scale: No; Yes, on rare occasions; Yes, almost every day; Yes, at least once a day; Yes, several times a day). Throughout the following waves (2-4), the questions addressed tobacco use within the past 12 months and were answered with the same five ordinal scale as described above. For this study, the variables were recoded into three grade ordinal scales with following categories: *No*, *Occasional* and *Regular* (including almost daily and daily use). For some analyses, a dichotomous outcome of tobacco use (yes/no) was used, i.e. individuals reporting any use ever (W1) or within the past 12 months (W2-W4). *Users* were defined as using any tobacco product, i.e. cigarettes, snus or both, while *dual users* were defined as using both cigarettes and snus. When categorized, the group “*cigarette smoker*” could thus also include individuals that were dual users. Thus, in sub analysis, we also separated data into *exclusive users*, that were defined as using either cigarettes or snus only, i.e. conditioned that they did not report use of the other product.

##### Confounders

In the logistic regressions we opted to adjust for specific factors, including the adolescent’s perception of their economic status relative to other families, gender, previous inebriation, novelty seeking, and tobacco use at W4. Socioeconomic status was assessed by the following question: *How do your family’s finances compare to other families where you live?* “We have less money”, “We have as much”, “We have more money”. Novelty seeking was measured using the Swedish version of the Junior and Temperament Character Inventory (JTCI), which previously has been validated in a Swedish context.^44,45^ Since the JTCI question battery is extensive (108 items), it was only performed once during W2 (α = 0.68). The individuals’ legal gender was used in the analyses. Demographics of the confounders are presented in Table 1.

#### Outcome variables

##### Alcohol use

Variables measuring alcohol consumption (W1-W3) and inebriation (W1-W4) within the past 12 months had a six-grade ordinal scale [No (0); Once (1); Several times (2); Once a month (3); 2-3 times a month (4); Once a week or more often (5)]. Alcohol consumption in W4 was assessed using the Alcohol Use Disorders Identification Test (AUDIT).^46^ Alcohol consumption was categorized as: Never (0), Once a month or less often (1), 2-3 times a week (2), and Four times a week or more often (3). In the descriptive statistics, the categories were combined into: *Occasional*, which included “once” or “several times” (W1-W3) or “Once a month or less often (W4)], and *Regular,* which included “once a month”, “2-3 times a month” and “once a week or more often” (W1-W3) or “2-3 times a week” and “Four times a week or more often” (W4). To outline transitions in alcohol use in relation to nicotine initiation, the relative proportion of individuals reporting drinking alcohol regularly (more than once a month) was descriptively presented in Figure 3.

At W4, adolescents were assessed for hazardous alcohol use, using AUDIT.^46^ To determine hazardous alcohol use we utilized AUDIT-C (α = 0.86), the first three questions of AUDIT. Both AUDIT and AUDIT-C are validated in a Swedish context,^47^ and a cut-off score of three or higher on AUDIT-C indicates at-risk alcohol consumption.^48^ Prospective associations between tobacco use and at-risk consumption was assessed using logistic regression, for which five models were constructed: An unadjusted model only containing tobacco use at W3, Model A [adjusted for gender and self-rated socioeconomic status (SES)], Model B (adjusted for gender, SES, and inebriation at W3), Model C [adjusted for gender, SES, inebriation at W3, and novelty seeking (NS)], and Model D (adjusted for gender, SES, inebriation at W3, NS, and tobacco use at W4).

### Statistical analysis

For comparisons between different groups, chi-square test, independent samples *t* test, and independent samples median test were used when applicable. Effect sizes were calculated utilizing Cohen’s d for t-tests and Phi coefficient or Cramer’s V for chi-square tests. Post hoc pairwise comparisons were made using Bonferroni adjusted alpha levels. Correlations between ordinal variables were assessed using Spearman´s ρ. Mixed analysis of variance was used to model transitions in alcohol use over time. The binomial test was used to assess gender distribution in each wave.

To investigate the postulated role of tobacco as a risk factor for hazardous alcohol consumption at W4, we employed binary logistic regression, controlling for various confounders. The logistic regressions were assessed for multicollinearity and the model’s predictive capability (chi-square test and Hosmer-Lemeshow analysis) to ensure robustness. Only participants providing a response to all the variables of interest during one specific wave were included in the analyses. Data are presented as mean values ±95% confidence interval (CI) unless anything else is clearly stated.

## Results

### Demographics

Demographics of the study participants for each wave are presented in Table 1. Out of 2108 individuals originally invited, 1515 responded to the initial survey at W1. The distribution between genders did not differ in W1-W3, but at W4 there were significantly more girls participating (*P* < 0.001). Most study participants reported their family’s socioeconomic status as having “*as much money*” as other families. Novelty seeking was measured at W2 and differed between genders, with boys scoring higher compared to girls [t (1386) = 3.30, *P* < 0.001)]. Over the last two waves, individuals lost to follow up was significantly higher among tobacco users [ 28% vs 18%, χ^2^_(1)_ = 12.62 *P* < 0.001 (W2-W3); 54% vs 40%, χ^2^_(1)_ = 14.85, *P* < 0.001 (W3-W4)] (Table 1).

### Tobacco use over the study period

**Figure 1.**
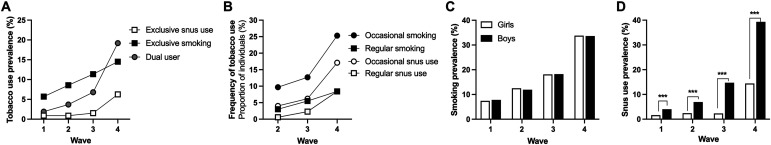
Tobacco use over the study period. (A) Tobacco use increased over the study period. Smoking was more common than snus use, and dual use was common among snus users. (B) Occasional use of cigarettes or snus was more common than regular use (daily or almost daily). (C–D) Smoking prevalence did not differ between boys and girls, but snus use was more common among boys. Data are percentage and based on 949-1515 responses. **P < .01, ***P < .001.

At baseline (W1), 8.5 percent reported use of any tobacco product, with 2.3 percent reporting dual use (Figure 1A). The number of individuals that had used tobacco increased over time (Figure 1A), and occasional use of cigarettes or snus was more common than regular use (Figure 1B). At W4, when respondents were around seventeen years old, 40 percent reported use of any tobacco product during the past year. Exclusive use of snus was rare throughout W1-W3 but increased in W4 (Figure 1A). Smoking prevalence at W1 did not differ significantly between genders (7.4% of girls vs 7.8 % of boys, χ^2^_(1)_ = 0.10, *P* = 0.750) (Figure 1C), but it was more common that boys used snus compared to girls (4.0% vs 1.6%, χ^2^_(1)_ = 7.20, *P* = 0.007, Cramer´s V = 0.071) (Figure 1D).

### Alcohol use over the study period

**Figure 2.**
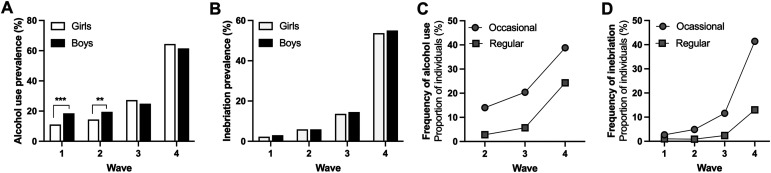
Alcohol use over the study period. (A) Having consumed alcohol was more common among boys in the first two waves, but not in W3 and W4. (B) Inebriation was independent on sex and increased over the study period. (C–D) It was more common to use alcohol or be inebriated sporadically (once or a few times), compared to regularly (monthly or weekly). Data are percentage and based on 949-1515 responses. **P < .01, ***P < .001.

During W1, 14.5 percent of the respondents reported ever use of alcohol. The proportion of individuals reporting alcohol use increased over time (Figure 2A), and at W4, more than half of the study population [n = 597 (63.1%)] had consumed alcohol over the past year. Having tried alcohol was more common among boys in W1 (18.5 % vs 11.1%, χ^2^_(1)_ = 15.70, *P* < 0.001, Cramer´s V = 0.11) and W2 (19.5 % vs 14.4%, χ^2^_(1)_ = 6.73, *P* = 0.010, Cramer´s V = 0.068), but no significant differences were detected in W3 and W4 (Figure 2A).

Throughout all waves, boys and girls reported inebriation to a similar extent, and the proportion of respondents reporting inebriation over the last 12 months increased over time (Figure 2B). In W4, 54.4 percent (n = 512) had been inebriated within the past 12 months. It was more common to have used alcohol or been inebriated sporadically compared to regularly (once a month or more often) (Figure 2C–D).

### Early and late onset of tobacco use in relation to alcohol consumption

**Figure 3.**
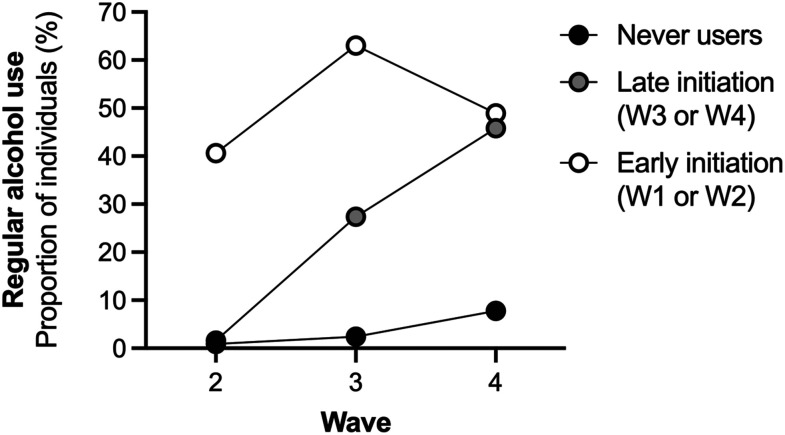
Tobacco initiation is associated with a transition in alcohol use. Regular alcohol use (once a month or more) was common among individuals that were tobacco users during the first or second wave throughout the study period. Individuals that initiated their tobacco use during W3 or W4 had a similar frequency of alcohol consumption as never users during W2, but then transitioned to the same level as early initiators at W4. Data are mean values and based on 753-867 responses.

Having tried alcohol at W1 was more common among individuals that reported use of tobacco, compared to those who did not (68.6% vs 9.6%, χ^2^_(1)_ = 308.83, *P* < 0.001, Cramer´s V = 0.47). To further assess the role of debut of tobacco initiation in relation to frequency of alcohol consumption, tobacco users were subdivided into *early initiators* (using tobacco at W1 and/or W2; n = 71) and *late initiators* (not using at W1 or W2 but using at W3 and/or W4; n = 230). Ever tobacco users were further compared to the group *never tobacco user* (not using tobacco in any wave, n = 332). Regular use of alcohol was uncommon during W2 among never tobacco users and late initiators, but relatively common among tobacco users (Figure 3). When categorizing data based on the frequency of alcohol use, alcohol consumption increased over the study period in all groups (mixed ANOVA F _(1, 630)_ = 326.71, *P* < 0.001), but there was a significant difference between the three groups (F _(2, 630_) = 205.53, *P* < 0.001). There was also a significant interaction between the different groups (F _(2,630)_ = 59.19, *P* < 0.001), as *late initiators* transitioned from displaying a relatively low frequency of alcohol use to more regular use (Figure 3).

### Prospective association between tobacco use in W3 and at-risk consumption in W4

In the next set of analysis, logistic regression was used to assess the prospective association between tobacco use at W3 and at-risk alcohol consumption (AUDIT-C ≥3 points) at W4. Use of any tobacco product in W3 was significantly associated with increased odds for at-risk consumption of alcohol at W4 (Table 2). Tobacco use at W3 maintained a risk factor for future at-risk consumption after adjusting for gender, socioeconomic status, inebriation at W3, and novelty seeking (model A-C, Table 2). However, in the fully adjusted model D, including tobacco use at W4, use of tobacco in W3 was no longer a significant predictor for at-risk consumption of alcohol at follow up (OR = 1.34, CI 95: 0.69-2.58, *P* = 0.389). Separating respondents based on the tobacco product used, cigarette smoking in W3 was associated with increased odds for AUDIT-C score above 3 in model A-C, whereas use of snus was a significant predictor in the unadjusted model and model A, but not in models B-D (Table 2). Individual contribution to the odds ratio of hazardous alcohol use for the exposure variables is outlined in Supplemental Table 2A-D.

**Table 2.** Association Between Tobacco Use in W3 and At-Risk Alcohol Consumption in W4. Odds Ratios With 95% CI for Different Forms of Tobacco Use in W3 in Relation to at-Risk Consumption of Alcohol in W4.

Wave 3	Unadjusted		Model A		Model B		Model C		Model D	
	OR (CI)	*P*	OR (CI)	*P*	OR (CI)	*P*	OR (CI)	*P*	OR (CI)	*P*
Any tobacco use	6.01 (3.71-9.73)	<0.001	5.97 (3.68-9.70)	<0.001	3.53 (2.09-5.95)	<0.001	2.92 (1.64-5.19)	<0.001	1.34 (0.69-2.58)	0.389
Any cigarette smoking	7.03 (4.19-11.81)	<0.001	6.97 (4.14-11.72)	<0.001	4.08 (2.33 - 7.13)	<0.001	3.14 (1.71-5.78)	<0.001	1.55 (0.77-3.11)	0.216
Any snus use	5.29 (2.42 -11.57)	<0.001	5.52 (2.49-12.26)	<0.001	2.10 (0.86-5.13)	0.103	1.97 (0.71-5.52)	0.195	0.84 (0.29-2.42)	0.539
Dual use	9.68 (3.39-27.66)	<.001	9.98 (3.45-28.84)	<.001	3.22 (1.02-10.19)	<.001	2.48 (0.67-9.24)	0.175	1.23 (0.32-4.83)	0.764

A: adjusted for gender and self-rated socioeconomic status (SES). B: adjusted for gender, SES, and inebriation in W3. C: adjusted for gender, SES, inebriation in W3, and novelty seeking. D: adjusted for gender, SES, inebriation in W3, novelty seeking, and tobacco use in W4.

### Association between tobacco use over the last year and alcohol consumption at W4

Since results from logistic regression analysis suggested that tobacco use within the past year (W4) had a larger impact on the odds for at-risk alcohol consumption compared to earlier use (W3), and the number of exclusive snus users increased dramatically during W4, detailed comparisons of different aspects of tobacco use in W4 were further analyzed in relation to AUDIT-C scores.

**Figure 4.**
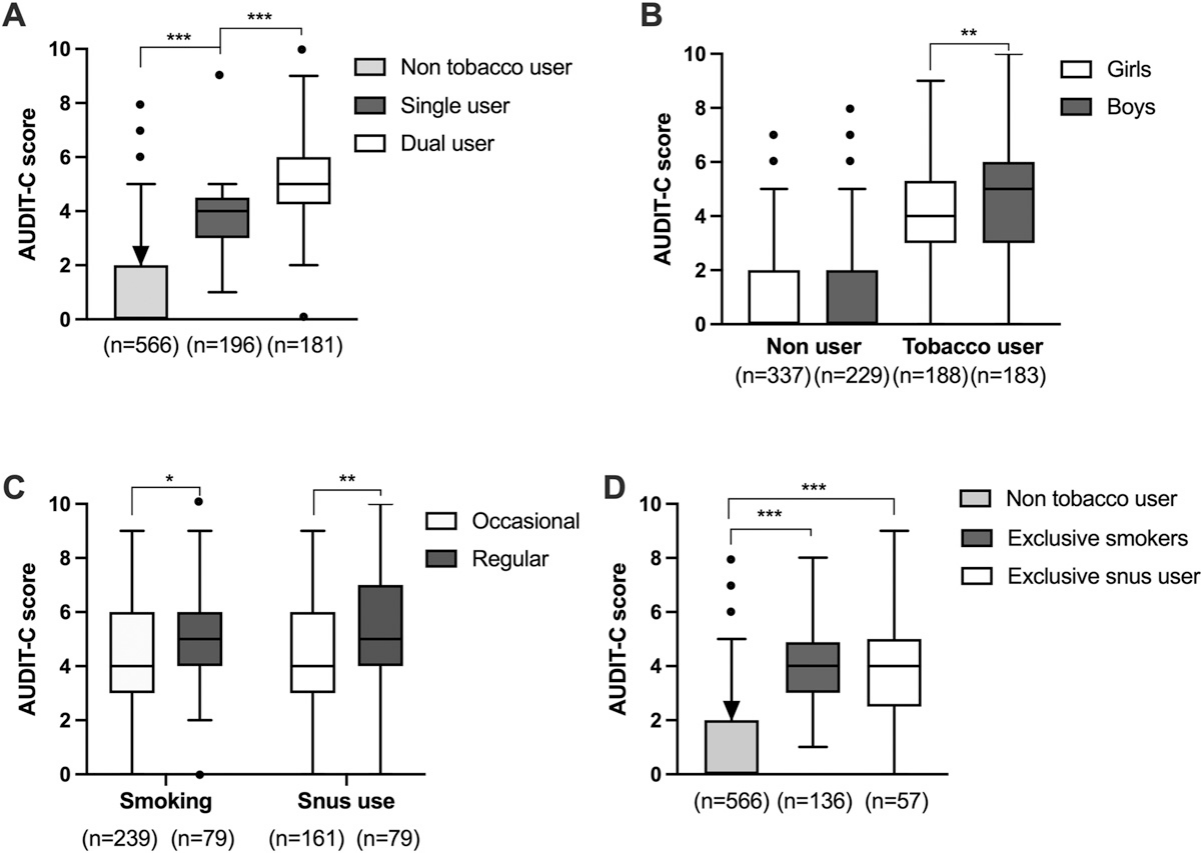
Tobacco use is associated with higher AUDIT-C scores independent on product used. (A) Tobacco users, and especially dual users, demonstrated increased AUDIT-C scores compared to non-tobacco users. (B) Boys using tobacco scored higher on AUDIT-C compared to female users, but no gender difference was detected among non-users. (C) Regular use of cigarettes or snus is associated with higher AUDIT-C scores compared to occasional use. (D) Both exclusive smoking as well as exclusive snus use over the last 12 months was associated with at-risk consumption of alcohol. n = number of individuals in the corresponding categories. *P < .05, **P < .01, ***P < .001.

Tobacco use significantly increased median AUDIT-C score (median 4 vs 0, *P* < 0.001), and individuals reporting dual use scored higher compared to single users (Bonferroni post hoc: nonuser vs single user: *P* < 0.001; single user vs dual user, *P* < 0.001) (Figure 4A). Sub-analysis further revealed that boys using tobacco products had a higher median AUDIT-C score compared to girls (median 5 vs 4, *P* = 0.003) (Figure 4B). Both smoking and snus use was associated with high AUDIT-C score, and regular smoking or snus use was associated with higher AUDIT-C scores compared to occasional smoking (*P* = 0.016) or snus use (*P* = 0.005) (Figure 4C).

Out of the 240 individuals reporting snus use in W4, 75.4 percent also reported smoking. Since dual use was common among snus users, a detailed analysis was performed looking more selectively at individuals reporting exclusive use of either product compared to non-tobacco users. Exclusive use of cigarettes was associated with higher AUDIT-C scores compared to non-tobacco users (*P* < 0.001), as was exclusive use of snus (*P* < 0.001) (Figure 4D). In fact, further statistical analysis demonstrated that exclusive smoking was associated with more than 10 times higher odds of at-risk alcohol consumption compared to non-tobacco use, whereas exclusive users of snus was associated with a nearly seven times increase (Table 3). After adjusting for gender, SES, and inebriation in W3, both exclusive use of cigarettes and exclusive use of snus were still significantly associated with increased odds of at-risk consumption (Table 3).

**Table 3.** Association Between Tobacco Use in W4 and At-Risk Alcohol Consumption in W4. Odds Ratios With 95% CI for Different Forms of Tobacco Use in W4 in Relation to at-Risk Consumption of Alcohol in W4.

Wave 4	Unadjusted		Model B	
	OR (CI)	*P*	OR (CI)	*P*
Cigarette smoking	10.68 (6.87-16.60)	<.001	6.94 (4.21-11.43)	<.001
Use of snus	6.85 (3.84-12.24)	<.001	5.39 (2.70-10.77)	<.001

B: adjusted for gender, SES, and inebriation in W3.

## Discussion

The aim of this study was to assess the association between tobacco use and alcohol consumption in youths with special emphasis on tobacco product used and gender. The data presented demonstrates that use of tobacco, independent of product used, is associated with a prospective increase in alcohol consumption. Tobacco use at W3 was a risk factor for at-risk alcohol consumption at W4 even when adjusting for alcohol inebriation at W3 and risk-taking behavior. Dual use, and the frequency of product use, further strengthened the association. Importantly, similar to never users, individuals initiating tobacco use at later waves (W3 or W4) had a low proportion of regular alcohol users at W2. However, upon tobacco initiation, a transition occurred resulting in as high levels of regular alcohol users as seen among early initiators at W4. Thus, our data supports a prospective association between tobacco initiation and increased alcohol use. These associations were further observed in both boys and girls. Considering the escalated use of non-combustible nicotine products among adolescents and young adults, these findings have important public health implications and highlight the need for policies designed to counteract all forms of nicotine use among youths.

While tobacco use in adolescence have been linked to hazardous alcohol intake,^7,17^ and data from e-cigarette users support these associations,^20,49,50^ little is known about the risk associated with smokeless tobacco such as snus, and dual use of snus and cigarettes. By analyzing exclusive use of snus and cigarettes in relation to alcohol consumption we showed that both products were independently associated with higher AUDIT-C scores, suggesting that nicotine is driving the association independent on route of administration. While snus use was relatively uncommon among girls over the first three waves, this proportion increased to 14 percent during W4. The increase in snus use among girls might reflect the introduction of nicotine pouches during the study period, since the questionnaires did not differentiate between the two products. Indeed, while Swedish snus is not that common internationally, it in many ways resembles nicotine pouches, which are now gaining popularity among adolescents.^51-54^ Snus use has previously been associated with increased risk for development of alcohol dependence in adults,^38^ and young male snus users have been shown to consume even more alcohol than smokers.^55^ Importantly, the data presented here demonstrated that dual use was associated with higher AUDIT-C scores compared to single use. Smokeless nicotine products may thus dispose a major public health issue, both by itself and by promoting dual product use, which may further increase the risk of hazardous alcohol intake among both boys and girls.^40,56^

During W1 and W2, it was more common for boys to have used alcohol, but during later waves boys and girls reported similar patterns of alcohol consumption. When novelty seeking behavior was measured at W2 boys scored significantly higher compared to girls, a phenomenon that has been described also in other samples.^44,57^ Due to the extensiveness of the JTCI inventory, no additional measurements of novelty seeking were made during later waves. It thus remains unknown if the difference persisted throughout the consecutive waves. Regardless, prevalence of alcohol use and inebriation did not differ between boys and girls at W3 and W4, and tobacco use remained a significant predictor for future at-risk consumption even after adjustment for novelty seeking. Tobacco use in W3 further remained a risk factor for high AUDIT-C score when adjusting for inebriation. While inebriation might be considered a mediation variable for high AUDIT-C scores, there is a complex relationship between alcohol and tobacco use patterns, where the order of progression is not universal and may reflect cultural factors.^58^ While tobacco use may be better at predicting subsequent alcohol use,^58^ smokers smoke more cigarettes while under the influence of alcohol, and report greater smoking satisfaction after alcohol.^59-61^ Importantly, adolescent smokers demonstrate increased vulnerability to AUD compared with nonsmokers who drink equivalent amounts of alcohol,^7^ suggesting that nicotine may increase AUDIT-C scores through alternative pathways. Putatively, the susceptibility to develop AUD is connected to the postulated influence by nicotine on brain circuits of importance for reward and cognitive control,^9,11-14^ thereby increasing the risk of addictive behavior.^62^

Considering that tobacco use is associated with gender differences among adults, we aimed to define if the association between tobacco use and alcohol was gender specific. While no difference in AUDIT-C score was detected between boys and girls in general, boys using tobacco scored higher compared to girls using tobacco. As exclusive snus use among young males previously has been linked to frequent binge drinking and higher alcohol consumption compared to smokers,^55^ the higher prevalence of snus use and dual use among males may explain parts of this association. Nonetheless, while tobacco use increased hazardous drinking in both boys and girls, there is a possibility that males might be especially vulnerable to the effects of nicotine on alcohol consumption. For instance, transdermal nicotine administration has been shown to increase alcohol intake in men, but not in women.^63^

Initiating tobacco use at an early age has repeatedly been associated with future alcohol misuse.^64-66^ When monitoring subgroups of tobacco users, we found that late initiators had a low proportion of regular alcohol users at W2, and that this proportion was similar to never users. However, upon tobacco initiation, this proportion progressively increased over the study period and reached similarly high levels as early initiators at W4. In addition, more frequent users with higher nicotine exposure displayed increased AUDIT-C scores compared to sporadic users and nonusers. These findings collectively support the idea of nicotine as a promoter for hazardous alcohol consumption. While the underlying mechanisms remain to be defined, nicotine may in the acute phase reverse the subjective sensations of intoxication and sedation,^67,68^ which in turn could increase the risk of engaging in binge drinking. Nicotine may also enhance the rewarding property of alcohol via both central and peripheral mechanisms involving the nAChR,^69-71^ thereby promoting excessive alcohol intake. Furthermore, repeated nicotine use may transform important neuronal circuits leading to heightened susceptibility to develop addictive behaviors.^62,72,73^ Additional studies are needed to further define the mechanism underlying the association.

The strength of this study lies in the longitudinal approach, where adolescents have been followed during a period in life where tobacco and alcohol use is initiated. By following adolescents during this timeframe, we could capture the transition from low alcohol consumption to high alcohol consumption in relation to tobacco initiation, and our data supports a prospective association. There are several weaknesses of this study. One weakness lies in that no power analysis was performed, and that the current sample includes a relatively low number of exclusive snus users, especially among girls, thereby reducing the strength of the statistical analysis. In addition, the attrition rate was high, which may have biased estimates of associations.^74^ Especially, tobacco users were lost to follow up to a greater extent compared to non-users. Hence, the effect size of the prospective association between nicotine exposure in W3 and alcohol consumption in W4 might have been underestimated. Furthermore, while the first waves were conducted one year apart, it was a longer duration between W3 and W4. Considering the fully adjusted model which controlled for tobacco use at W4, an even stronger association between tobacco use at W3 and at-risk consumption of alcohol at follow-up might would have been observed if the measuring points had been closer in time.

## Conclusion

In addition to the chronic effects that tobacco use has on morbidity later in life, the data presented here clearly demonstrate that both combustible and smokeless tobacco is associated with a prospective increase in hazardous alcohol intake, and that dual use further strengthens this association in both boys and girls. From a public health perspective, preventive measures to counteract the increase in smokeless nicotine products on the market, and policies designed to reduce all forms of nicotine use among youths is warranted.

## Supplemental Material

**Supplemental Material -** Prospective Association Between Tobacco Use and at-Risk Alcohol Consumption Among Swedish Adolescents: Outlining the Influence of Tobacco Product, Frequency of Use and Gender in the LoRDIA Cohort Supplemental Material for Prospective Association Between Tobacco Use and at-Risk Alcohol Consumption Among Swedish Adolescents: Outlining the Influence of Tobacco Product, Frequency of Use and Gender in the LoRDIA Cohort by Johanna Andersson, Kristina Berglund, Robin Irmel, and Louise Adermark in Tobacco Use Insights.

## Data Availability

The data underlying this article will be shared upon reasonable request to the corresponding author.*
