# Supplementary material for: Prospective Association Between Tobacco Use and at-Risk Alcohol Consumption Among Swedish Adolescents: Outlining the Influence of Tobacco Product, Frequency of Use and Gender in the LoRDIA Cohort
Source: Tob Use Insights. 2024 Oct 30;17:1179173X241298524. doi: 10.1177/1179173X241298524 (PMC11528605; doi:10.1177/1179173X241298524)
Supplement: Supplemental Material - Prospective Association Between Tobacco Use and at-Risk Alcohol Consumption Among Swedish Adolescents: Outlining the Influence of Tobacco Product, Frequency of Use and Gender in the LoRDIA Cohort [file sj-pdf-1-tui-10.1177_1179173X241298524.pdf]

Supplemental material: Prospective association between tobacco use and at-risk alcohol consumption among Swedish adolescents: Outlining the influence of tobacco product, frequency of use and gender in the LoRDIA cohort

Full analysis of table 2

Table 2A. Association between any tobacco use in W3 and at-risk alcohol consumption in W4

Odds ratios with 95% CI for different forms of tobacco use in W3 in relation to at-risk consumption of alcohol in W4.

	**Bivariate**		**Model A**		**Model B**		**Model C**		**Model D**	
**Wave 3**	*OR (CI)*	*p*	*OR (CI)*	*p*	*OR (CI)*	*p*	*OR (CI)*	*p*	OR (*CI*)	p
Any tobacco use	**6.01** (3.71-9.73)	<.001	**5.97** (3.68-9.70)	<.001	**3.53** (2.09-5.95)	<.001	**2.92** (1.64-5.19)	<.001	1.34 (0.69-2.58)	.389
Gender (ref girl)	1.18 (0.91-1.53)	.209	1.13 (0.83-1.54)	.448	1.19 (0.87-1.64)	.278	1.13 (0.80-1.60)	.494	0.93 (0.69-1.38)	.705
Self-rated economic status as others (ref)	1		1		1		1		1	
Less than others	0.96 (0.59-1.56)	.859	0.81 (0.44-1.48)	.493	0.79 (0.42-1.48)	.458	0.80 (0.41-1.58)	.521	0.68 (0.31-1.50)	.338
More than others	**1.46** (1.05-2.05)	.027	**1.66** (1.11-2.48)	.014	**1.71** (1.13-2.56)	.010	**1.58** (1.02-2.45)	.042	1.36 (0.83-2.24)	.228
Inebration last 12 months	**9.48** (4.94-18.19)	<.001			**5.53** (2.75-11.11)	<.001	**4.77** (2.27-10.01)	<.001	**2.98** (1.31-6.78)	.009
Novelty seeking	**1.26** (1.20-1.32)	<.001					**1.15** (1.09-1.22)	<.001	**1.13** (1.06-1.20)	<.001
Tobacco use in W4	**13.56** (9.84-18.69)	<.001							**9.71** (6.43-14.66)	<.001

A: adjusted for gender and self-rated socioeconomic status (SES)

B: adjusted for gender, SES, and inebriation in W3

C: adjusted for gender, SES, inebriation in W3, and novelty seeking

D: adjusted for gender, SES, inebriation in W3, novelty seeking, and tobacco use in W4.

**Table 2B. Association between any cigarette smoking in W3 and at-risk alcohol consumption in W4**<br>Odds ratios with 95% CI for different forms of tobacco use in W3 in relation to at-risk consumption of alcohol in W4.										
	**Unadjusted**		**Model A**		**Model B**		**Model C**		**Model D**	
**Wave 3**	*OR (CI)*	*p*	*OR (CI)*	*p*	*OR (CI)*	*p*	*OR (CI)*	*p*	OR (*CI*)	p
Any cigarette smoking	**7.03** (4.19-11.81)	<.001	**6.97** (4.14-11.72)	<.001	**4.08** (2.33-7.13)	<.001	**3.14** (1.71-5.78)	<.001	1.55 (0.77-3.11)	.216
Gender (ref girl)	1.18 (0.91-1.53)	.209	1.16 (0.85-1.59)	.342	1.22 (0.89-1.68)	.230	1.15 (0.81-1.62)	.439	0.93 (0.62-1.38)	.928
Self-rated economic status as others (ref)	1		1		1		1		1	
Less than others	0.96 (0.59-1.56)	.859	0.87 (0.48-1.58)	.640	0.86 (0.46-1.59)	.622	0.83 (0.43-1.63)	.595	0.70 (0.32-1.53)	.376
More than others	**1.46** (1.05-2.05)	.027	**1.62** (1.08-2.43)	.019	**1.68** (1.11-2.52)	.013	**1.56** (1.00-2.42)	.050	1.36 (0.82-2.24)	.231
Inebration last 12 months	**9.48** (4.94-18.19)	<.001			**5.28** (2.62-10.62)	<.001	**4.67** (2.22-9.81)	<.001	**2.82** (1.24-6.44)	.014
Novelty seeking	**1.26** (1.20-1.32)	<.001					**1.15** (1.09-1.22)	<.001	**1.13** (1.06-1.20)	<.001
Tobacco use in W4	**13.56** (9.84-18.69)	<.001							**9.66** (6.42-14.55)	<.001
A: adjusted for gender and self-rated socioeconomic status (SES)<br>B: adjusted for gender, SES, and inebriation in W3<br>C: adjusted for gender, SES, inebriation in W3, and novelty seeking<br>D: adjusted for gender, SES, inebriation in W3, novelty seeking, and tobacco use in W4.										

Table 2C. Association between any snus use in W3 and at-risk alcohol consumption in W4

Odds ratios with 95% CI for different forms of tobacco use in W3 in relation to at-risk consumption of alcohol in W4.

	**Bivariate**		**Model A**		**Model B**		**Model C**		**Model D**	
**Wave 3**	*OR (CI)*	*p*	*OR (CI)*	*p*	*OR (CI)*	*p*	*OR (CI)*	*p*	OR (*CI*)	p
Any snus use	**5.29** (2.42-11.57)	<.001	**5.52** (2.49-12.26)	<.001	2.10 (0.86-5.13)	.103	1.97 (1.71-5.51)	.195	0.78 (0.15-4.01)	.767
Gender (ref girl)	1.18 (0.91-1.53)	.209	1.01 (0.74-1.37)	.956	1.16 (0.84-1.60)	.364	1.09 (0.77-1.55)	.614	0.94 (0.63-1.40)	.767
Self-rated economic status as others (ref)	1		1		1		1		1	
Less than others	0.96 (0.59-1.56)	.859	0.75 (0.41-1.35)	.334	0.74 (0.40-1.39)	.352	0.73 (0.37-1.44)	.367	0.68 (0.31-1.49)	.337
More than others	**1.46** (1.05-2.05)	.027	**1.73** (1.17-2.56)	.006	**1.75** (1.17-2.62)	.006	**1.58** (1.02-2.44)	.040	1.34 (0.82-2.21)	.247
Inebration last 12 months	**9.48** (4.94-18.19)	<.001			**8.22** (4.16-16.27)	<.001	**6.33** (3.06-13.10)	<.001	**3.42** (1.50-7.83)	.004
Novelty seeking	**1.26** (1.20-1.32)	<.001					**1.17** (1.11-1.24)	<.001	**1.14** (1.07-1.21)	<.001
Tobacco use in W4	**13.56** (9.84-18.69)	<.001							**10.23** (6.83-15.32)	<.001

A: adjusted for gender and self-rated socioeconomic status (SES)

B: adjusted for gender, SES, and inebriation in W3

C: adjusted for gender, SES, inebriation in W3, and novelty seeking

D: adjusted for gender, SES, inebriation in W3, novelty seeking, and tobacco use in W4.

Table 2D. Association between dual use in W3 and at-risk alcohol consumption in W4

Odds ratios with 95% CI for different forms of tobacco use in W3 in relation to at-risk consumption of alcohol in W4.

	**Bivariate**		**Model A**		**Model B**		**Model C**		**Model D**	
**Wave 3**	*OR (CI)*	*p*	*OR (CI)*	*p*	*OR (CI)*	*p*	*OR (CI)*	*p*	OR (*CI*)	p
Dual use	**9.68** (3.39-27.66)	<.001	**9.98** (3.45-28.84)	<.001	**3.22** (1.02-10.19)	<.001	2.48 (0.67-9.24)	.175	1.23 (0.32-4.83)	.764
Gender (ref girl)	1.18 (0.91-1.53)	.209	1.02 (0.75-1.39)	.897	1.16 (0.84-1.60)	.368	1.10 (0.78-1.56)	.588	0.92 (0.62-1.38)	.699
Self-rated economic status as others (ref)	1		1		1		1		1	
Less than others	0.96 (0.59-1.56)	.859	0.73 (0.40-1.34)	.310	0.74 (0.40-1.38)	.343	0.73 (0.37-1.43)	.350	0.66 (0.30-1.45)	.303
More than others	**1.46** (1.05-2.05)	.027	**1.70** (1.15-2.52)	.008	**1.73** (1.16-2.59)	.007	**1.56** (1.01-2.41)	.045	1.35 (0.82-2.22)	.238
Inebration last 12 months	**9.48** (4.94-18.19)	<.001			**7.68** (3.87-15.22)	<.001	**6.17** (2.97-12.80)	<.001	**3.15** (1.37-7.27)	.004
Novelty seeking	**1.26** (1.20-1.32)	<.001					**1.17** (1.11-1.24)	<.001	**1.13** (1.06-1.21)	<.001
Tobacco use in W4	**13.56** (9.84-18.69)	<.001							**10.08** (6.73-15.10)	<.001

A: adjusted for gender and self-rated socioeconomic status (SES)

B: adjusted for gender, SES, and inebriation in W3

C: adjusted for gender, SES, inebriation in W3, and novelty seeking

D: adjusted for gender, SES, inebriation in W3, novelty seeking, and tobacco use in W4.
